# The Impact of Childhood Maltreatment on the Development of Borderline Personality Disorder in Adolescents: A Narrative Review

**DOI:** 10.7759/cureus.99736

**Published:** 2025-12-20

**Authors:** Mustafa A Alhammad, Hayat M Alshammari, Nouf W Alnafea, Kady S Althunayyan, Walaa S Mohammed, Manar M Almohamadi

**Affiliations:** 1 Internal Medicine, King Abdulaziz University, Jeddah, SAU; 2 Psychiatry, Irada Mental Health Hospital, Qassim, SAU; 3 Psychiatry, King Saud University Medical City, Riyadh, SAU; 4 Medicine, Qassim University, Buraydah, SAU; 5 Psychiatry, Al-Dammam Medical Complex, Al-Dammam, SAU; 6 Psychiatry, King Abdulaziz University, Jeddah, SAU

**Keywords:** adolescents, adverse childhood experience, borderline personality disorder, childhood maltreatment, emotional dysregulation, etiopathogenesis, mediator, neglect

## Abstract

The influence of early life trauma on the emergence of borderline personality symptoms has been extensively researched. However, despite the established correlation between childhood maltreatment and borderline personality disorder (BPD), the specific types of trauma and the mediating pathways remain insufficiently mapped. While sexual abuse has received substantial attention due to its measurable clinical impact, the roles of withdrawal and emotional neglect lack early detection and intervention during childhood. Understanding the multifactorial origins of BPD is essential for developing early preventive strategies and effective therapeutic models. This review discusses how the multiple and cumulative effects of maltreatment, including omission-type maltreatment such as emotional neglect, may contribute to the development of BPD through both physical and psychological mediators. This narrative review emphasizes the necessity of recognizing invisible or under-addressed trauma types, proposing conceptual pathways, and informing trauma-informed care focused on the physical, emotional, and social safety of children and adolescents.

## Introduction and background

Actions of aggression

Borderline personality disorder (BPD) is a psychiatric condition marked by emotional dysregulation, impulsivity, unstable relationships, and self-identity disturbances [1]. Individuals with BPD often struggle with emotional instability, exhibiting extreme emotional reactions and fluctuating self-concepts. According to the World Health Organization, one in eight individuals worldwide suffers from a mental disorder [2].

Despite the growing awareness of mental health issues, BPD remains underrepresented in epidemiological surveys across all genders, particularly among children and adolescents, due to diagnostic intricacies, symptom overlap with other disorders, and developmental factors. However, the fact that BPD symptoms appear early in adolescence, a critical period for identity, social, and emotional development, emphasizes the necessity for early identification, intervention, and treatment [3].

Early development: Triggers and drivers (action of perpetuation)

Environmental stressors, early attachment experiences, neurobiological vulnerabilities, and genetic predispositions dynamically interact to contribute to BPD development. Insecure or disorganized attachment, early emotional trauma, and ongoing invalidation in the caregiving environment are major factors [3-5]. In predisposed individuals, minor stressors such as academic pressure, peer rejection, or inconsistent parenting may exacerbate developing symptoms [4-6]. Neurodevelopmentally, adolescents with BPD frequently show structural and functional changes in the amygdala, prefrontal cortex, and hippocampal regions, which are linked to regulation and impulse control [7].

Role of childhood trauma as a mediator (action of withdrawal)

Childhood trauma, including emotional, physical, and sexual abuse, neglect, and bullying, is consistently identified as a strong predictor of BPD. A meta-analysis by Porter et al. (2020) found that up to 90% of individuals with BPD report childhood maltreatment [8]. Among these, childhood sexual abuse (CSA) often correlates with self-harm, emotional instability, and dissociation [5]. These experiences alter foundational self-and-other schemas, impairing emotional regulation and interpersonal functioning [9]. In hostile or invalidating environments, children may adopt maladaptive coping strategies such as avoidance or emotional suppression [10].

Early symptoms of BPD: Theoretical viewpoint

Albert Bandura’s Social Learning Theory suggests that children learn behaviors including emotion dysregulation and maladaptive interpersonal strategies not only through direct reinforcement, but also via modeling, imitation, and observation [11]. Children exposed to emotional instability, aggression, or neglect may internalize dysfunctional behaviors as normative, reinforcing core BPD features like emotional dysregulation and interpersonal difficulties. Conversely, supportive environments with safe attachments and positive role models may serve as protective factors.

Research gaps and review objective

Although the link between early adversity and BPD is well-supported, questions remain regarding developmental timing, causal pathways, and mediating psychosocial or neurobiological mechanisms. The differential effects of trauma types (e.g., abuse vs neglect) and the role of resilience, temperament, and social support are poorly understood [3-5]. This review aims to synthesize the evidence from empirical literature observational studies, meta-analyses, and systematic reviews on the impact of specific childhood adversities (bullying, neglect, emotional, physical, and sexual abuse) on adolescent BPD development and maintenance. The secondary objective is to inform prevention and early intervention by identifying mediators and moderators influencing developmental trajectories.

Method

This review followed a narrative approach to explore how different forms of childhood maltreatment may contribute to the development of BPD in adolescents. Between July and November 2024, we conducted a literature search across major databases including the Saudi Digital Library (SDL), PubMed, SCOPUS, and Google Scholar.

The search focused on English-language publications addressing the relationship between childhood trauma (physical, sexual, emotional abuse, bullying, neglect) and BPD features in children or adolescents. Study types included clinical guidelines, retrospective studies, case reports, and observational research. Articles were selected based on their relevance to the review topic, especially those identifying mediators or conceptual mechanisms linking trauma to early BPD development. No formal inclusion/exclusion protocol was applied, which aligns with this review's narrative nature. We developed a conceptual framework by synthesizing common patterns identified across the selected literature.

## Review

Action of aggression

Sexual Abuse

Sexual abuse refers to non-consensual sexual activity, including coercion, sexual assault, and exploitation, which can affect individuals of any age, gender, or background [12]. Sexual harassment includes various forms of verbal abuse (e.g., inappropriate comments), visual exposure (e.g., sexual images), online harassment (e.g., offensive posts), and physical assault (e.g., unwanted touching) [13].

Sexual abuse and harassment are deeply rooted in multifactorial determinants that require comprehensive, multidisciplinary solutions. Beyond acknowledging the acts of abuse themselves, it is essential to consider the broader sociocultural and developmental contexts in which they occur. Evidence indicates that risk factors begin as early as childhood and may be influenced by the home environment, exposure to violence, lack of psychosocial support, and limited access to education and mental health services. Effective responses must therefore include integrated strategies such as early psychological counseling within educational institutions, parental education programs, and targeted community outreach, including mobile mental health services for high-risk or underserved populations. Moreover, public health and educational policies must promote collaboration among healthcare providers, educators, legal systems, and social services to foster protective environments for children and adolescents [14].

Research consistently demonstrates a strong link between CSA and the development of BPD, with clinical studies indicating that individuals exposed to physical or sexual abuse are at an increased risk of developing BPD symptoms, such as identity disturbances, recurrent suicidal behaviors, self-harm, and dissociative reactions triggered by stress [15]. 

The severity and chronicity of CSA, mainly when perpetrated by family members, significantly influence the intensity of BPD symptoms. CSA survivors frequently turn to coping mechanisms like substance abuse, dissociation, or self-harm, further exacerbating emotional dysregulation. Additionally, CSA contributes to the development of negative core beliefs like shame and worthlessness, compounding emotional distress and relational challenges, such as fear of abandonment and unstable relationships. The absence of social support and adverse environmental factors can intensify the long-term psychological effects of CSA, creating unique challenges for treatment [13].

Scholars propose expanding the definition of CSA to include non-contact victimization, such as exposure to pornography or sexually suggestive material, given the profound psychological consequences these experiences can have on victims [16]. An exploratory study has also highlighted that sexual harassment or CSA shares a significant factor in the onset of BPD. These effects include long-lasting disruptions to emotional regulation and identity formation, which often worsen existing vulnerabilities to BPD [17].

Physical Abuse 

Physical abuse, defined as inflicting bodily harm in the form of strangulation, burns, welts, bruises, or broken bones, represents a significant risk factor for psychological disturbances. About 30-90% of individuals with BPD report histories of trauma, with physical abuse being one of the most prevalent forms [18]. Compared to non-abused children, physically abused children tend to have higher scores of emotional dysregulation, identity diffusion, interpersonal difficulties, and self-harm behaviors [18]. 

Another cross-sectional study estimated the lifetime prevalence of physical abuse to be approximately 22.6%, based on self-reports and parental accounts of childhood maltreatment [19]. Physical abuse during childhood has been associated with increased risk of long-term challenges in mental health, physical well‑being, and socio‑psychological adjustment [20].

Although childhood adversity in general is more frequent among adolescents with BPD than among clinical controls, the specific association between physical abuse and early-onset BPD appears inconsistent. In a multivariate model adjusting for other forms of abuse and family factors, only sexual abuse, along with low maternal care and dysfunctional family functioning, remained independent predictors of adolescent BPD, while physical abuse did not [21]. In a longitudinal follow-up study, physical abuse did not significantly differ between adolescents who retained a BPD diagnosis and comparison subjects, suggesting that additional contributory factors may play a role [22]. 

Despite some inconsistencies, multiple studies consistently link childhood physical abuse to increased emotional instability, relational challenges, heightened emotional distress, and more severe BPD symptomatology [23], (Doctoral Thesis: Trentacosti NL. Importance of Childhood Maltreatment on Borderline Personality in Adults: Meta-analysis; 2021). Adolescents from abusive backgrounds often adopt maladaptive strategies to seek intimacy and reduce fears of rejection or abandonment, aligning closely with attachment theory [24]. 

An integrated perspective suggests that the timing and chronicity of physical abuse may determine its impact on BPD development. Early sustained abuse can profoundly disrupt critical emotional processes and attachment systems, creating vulnerabilities to BPD symptomatology. Conversely, later abuse may amplify pre-existing emotional and relational difficulties, intensifying mood swings, identity confusion, and emotional dysregulation [25]. Given the serious emotional and developmental consequences associated with childhood physical abuse, it is essential to implement evidence-based strategies aimed at prevention. Parenting programs that focus on improving emotional regulation, stress management, and non-violent discipline have shown significant success in reducing the risk of physical abuse [26]. Additionally, a public health approach that includes early home-visiting, parent education, and broader social support systems has been recommended to prevent maltreatment and mitigate its long-term psychological impact, including the development of BPD [27]. Embedding these strategies within community and clinical settings offers a practical and ethical path toward reducing the incidence of childhood physical abuse and its associated outcomes.

Emotional Abuse 

Emotional maltreatment is characterized by actions or omissions that harm a child’s emotional well-being, resulting in observable changes in behavior, affect, or cognition. A systematic review emphasized the profound effects of emotional abuse on the ability to manage dysregulated emotions [9].

Research demonstrates that emotional abuse has a stronger association with emotional dysregulation in adulthood than either physical or sexual abuse [28]. Among various childhood traumas, emotional abuse often occurs alongside other forms of maltreatment, such as sexual abuse. This type of abuse can disrupt the growth of emotional control and appropriate expression of feelings; emotional abuse results in dysfunctional emotional regulation [27,29].

Victims of emotional abuse often internalize negative self-perceptions, which can lead to heightened sensitivity to stress and a diminished ability to form stable relationships. This sense of defectiveness contributes to an intense fear of abandonment. Additionally, maladaptive emotional responses impair their capacity to function effectively in new environments [29,30]. A systematic review found a significant link between emotional abuse or neglect and maladaptive coping strategies, such as self-harm and substance abuse [31]. These coping strategies are needed to manage emotional distress rooted in abuse. 

That suggests the emotional neglect inherent in emotional abuse may exacerbate the onset of BPD, with both genetic and environmental factors playing pivotal roles in determining disorder severity.

Verbal Abuse 

Verbal abuse involves behaviors that inflict emotional harm through verbal interactions, including name-calling, severe criticism, humiliation, and derogatory remarks [25]. Research highlights its role in impairing emotional regulation and forming stable self-concepts, which are crucial for healthy relationships [18]. Verbal abuse has a long-term psychological impact, particularly when combined with other forms of abuse. Emotional dysregulation impairs the formation of a stable self-concept, increasing the likelihood of unstable relationships and impulsive behaviors as hallmark features of BPD [32]. 

Research indicates that children exposed to verbal maltreatment by caregivers are at a significantly heightened risk not only for BPD but also for other personality disorders, such as narcissistic, obsessive-compulsive, and paranoid personality disorders [18]. Multiple studies emphasize that verbal abuse is a significant contributing factor to BPD development, underscoring its long-term impact [18,25,32]. Therefore, verbal abuse amplifies pre-existing emotional and psychological weaknesses, solidifying its role as a critical risk factor in the development of the disorder.

Bullying 

Bullying, defined as aggressive behavior involving power imbalances, significantly contributes to the development of BPD traits [33]. This form of maltreatment can manifest through verbal and physical aggression or more indirect behaviors such as social ostracism [34].

Bullying exacerbates long-term vulnerabilities and disrupts emotional regulation, identity formation, and interpersonal relationships, which further exacerbate BPD symptoms [9]. Victims of bullying often experience intense feelings of anger, diminished trust, and self-worth. Research consistently indicates that bullying during childhood or adolescence serves as a strong predictor for the early development of BPD traits [35,36]. Social ostracism, a form of bullying, fosters isolation, erodes self-esteem and reinforces maladaptive coping strategies that contribute to BPD’s persistence [37].

Action of perpetuation and withdrawal

Neglect

Neglect, usually defined as inadequate physical or emotional care, is a significant risk factor in the development of BPD and appears to play a more complex role in its onset than previously recognized [38,39]. Emotional neglect, in particular, has emerged as a clear pathway to BPD, with one study reporting an odds ratio of 17.73 for developing BPD symptoms among those exposed [8]. In addition, a sample study (60 participants) found that emotional neglect had a larger effect than emotional abuse, referring to the statistical strength of its indirect impact on emotional dysregulation via attachment anxiety. While physical neglect contributes to BPD, emotional neglect, often overlooked, can have even more profound effects on emotional dysregulation, a core feature of BPD [40].

Emerging evidence indicates that childhood neglect leads to cognitive and behavioral disturbances disrupts self-concept formation, and shapes expectations of future emotional experiences [41,42]. Neglect actively reshapes the developing brain’s emotional architecture rather than merely delaying emotional growth. This finding highlights how the absence of parental warmth can foster the maladaptive coping mechanisms observed in BPD [43]. 

Furthermore, neglect, when combined with certain temperament traits (such as high harm avoidance) and deficient parental care, is associated with later personality dysfunction [44]. Notably, ignoring a child’s negative emotions during formative years has been linked to the emergence of BPD features and significant difficulties in emotional regulation [45]. 

Childhood neglect also fosters enduring challenges in emotional regulation by impairing the processing of negative emotions and creating a persistent sense of emotional invalidation. While the link between emotional abuse and BPD is well-established [46], only recently has attention turned to how emotional neglect shapes BPD’s neurobiological underpinnings. Emerging evidence suggests that emotional neglect, especially when combined with other forms of trauma, severely undermines the development of adaptive emotional coping strategies. 

Emotional neglect is also strongly correlated with later mental health problems in high-risk individuals, with impaired personality functioning mediating this relationship [47]. Additionally, individuals with BPD often exhibit poor interpersonal boundaries and reduced interoceptive accuracy (diminished awareness of their physical and emotional states), which further impairs their emotional regulation and interpersonal functioning [48] 

The early identification of neglect, along with heightened awareness and the implementation of targeted interventions, is essential in mitigating its long-term psychological impact. Trauma-informed therapeutic approaches, such as Dialectical Behavior Therapy (DBT) and Mentalization-Based Treatment (MBT), have demonstrated potential in addressing emotional trauma and improving relational functioning in individuals diagnosed with BPD. Acknowledging neglect as a significant risk factor may contribute to a paradigm shift in both public perception and clinical practice, fostering more proactive prevention strategies and empathetic care for those affected [49].

Neurobiological changes related to neglect and their implications for emotional dysregulation 

MRI studies show that early neglect, particularly maternal detachment, is linked to reduced gray matter volume in critical emotion-regulating brain regions such as the amygdala and hippocampus [21]. This finding reframes neglect as a psychological phenomenon that fundamentally alters neural structures involved in emotion regulation. Neglect may also sensitize the amygdala to perceived abandonment, leading to hyperactive stress responses and the intense emotional reactivity characteristic of BPD. In essence, early neglect can “program” the brain for long-term emotional dysregulation, contributing to persistent BPD symptoms. These neurobiological insights underscore the need for holistic treatment approaches that integrate psychological interventions with strategies for neurobiological recovery to foster emotional resilience [50].

Conceptual framework

This review confirms a clear association between childhood maltreatment and the development of BPD. Maltreatment can be viewed as a continuum, ranging from overt aggression to more subtle forms of harm like neglect. Aggressive forms of trauma (e.g., physical violence, verbal abuse, bullying, sexual exploitation) elicit hypervigilance and a heightened anticipation of threat, which can serve as mediators between observable maltreatment and emotional abuse outcomes. 

By contrast, withdrawal represents subtler, often concealed forms of harm, including neglecting basic needs such as nourishment, social interaction, and caregiver engagement (both verbal and physical). Failure to provide appropriate structure, supervision, or guidance also falls within this category, contributing to environmental deprivation. 

**Figure 1:**
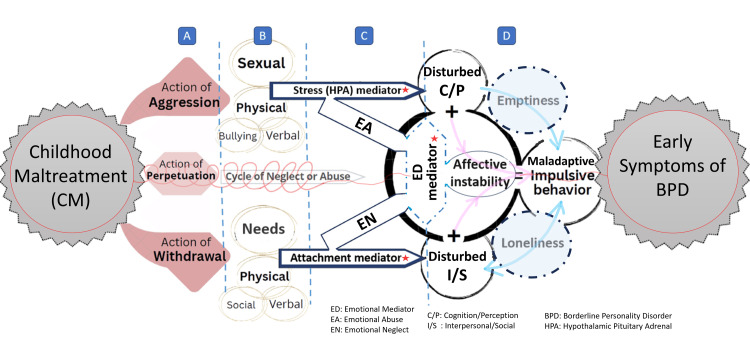
The pathway of early childhood maltreatment and contributing mediators to BPD development in adolescence A) Three key actions of maltreatment: aggression, perpetration, and withdrawal, illustrate its dynamic and reciprocal nature. Children may become aggressive with repeated exposure to abuse, and also withdraw emotionally in response to persistent neglect [33,51], (PhD dissertation. Banny A: Pathways From Child Maltreatment to Peer Functioning; 2015). The cyclical interaction between perpetrator and victim can sustain/amplify maltreatment, contributing to perpetuation of polytrauma [9]. B) Subtypes of aggression and withdrawal were ranked based on factors most strongly associated with BPD development [52], while also considering essential developmental needs, such as impact of impaired physical security (e.g., sexual attack) or unmet basic child needs [53]. Emotional abuse and neglect were separated due to distinct patterns of emergence, continuity, and long-term psychological impact [54]. C) Three mediators shape aggression and withdrawal. The stress mediator, reflected in hypothamalus pituitary adrenal (HPA) axis dysregulation, influences cognitive/perceptual disturbances, contributing to identity confusion and emotional dysregulation (ED) [55,56]. ED, whether under/over-regulated, is the most potent mediator of BPD symptoms, particularly affective instability (AI) [9,57]. The attachment mediator, anxious/avoidant, substantially disturbs interpersonal/social and further ED [40]. Attachment disruption, ED, and HPA-axis stress dysregulation jointly mediate the link between childhood maltreatment and early BPD, driving relational insecurity, AI, and heightened stress reactivity in adolescence. D) The “pink arrow” pathway represents four core BPD symptoms, while “blue arrow” reflects emptiness/loneliness, understood as a sequential state, where loneliness may progress to emotional emptiness [58]. Chronic emptiness reinforces social isolation through maladaptive behavior [59]. Image credit: Figure created using Microsoft PowerPoint (Microsoft Corp., Redmond, WA, USA).

The concept of perpetuation introduces another dimension of maltreatment, characterized by sustained and cumulative harm, which can persist into adolescence and adulthood. Perpetuation establishes cycles of abuse or neglect concept: A) While early instances of aggressive behavior often provoke immediate physiological threat responses, prolonged exposure causes anticipated forms of reactivity. B) Long-term environmental deprivation also mediates emotional neglect and the absence of warmth or emotional security, which profoundly affect emotional, cognitive, and behavioral impairments (Figure 1). 

Limitations

As a narrative review, our analysis is descriptive and subject to selection bias. The conceptual framework presented (Figure 1) is derived from literature synthesis and is intended for illustration only; it is not an empirically validated model. Future research should prospectively test these pathways and refine evidence-based prevention strategies.

Clinical implications

Children with maltreatment histories often show early borderline traits, particularly emotional dysregulation, unstable relationships, and identity issues. These findings stress the need for early trauma-focused screening and targeted interventions. Treatments like cognitive therapy, DBT, and mentalization-based approaches have shown promise. Applying these early to high-risk youth may prevent full BPD development and improve patients' symptoms.

Future research

Studies should improve trauma assessment tools and reduce methodological bias. A key priority is to elucidate how attachment disturbances (e.g. disorganized attachment) mediate or moderate BPD traits in adolescence. Similarly, research must dissect the influence of specific parenting styles - such as inconsistent caregiving, authoritarian discipline, or overinvolvement - distinguishing which parental factors exacerbate risk versus confer protection. Future research should focus on the neurodevelopmental aspects.

## Conclusions

Childhood maltreatment, particularly emotional neglect, physical abuse, and chronic sexual abuse, plays a critical role in the early development of BPD. The resulting disruptions to emotional regulation, identity, and relationships often persist into adolescence, with females disproportionately affected and at higher risk for severe symptoms and suicidality.

Addressing these outcomes requires early, trauma-informed interventions grounded in psychological frameworks such as Bandura’s social learning theory, which emphasizes self-efficacy and the role of supportive environments. Global organizations like the WHO, United Nations Children's Fund (UNICEF), and United Nations (UN) must prioritize ethically responsible strategies that protect children, promote resilience, and ensure long-term psychological well-being.

Given the profound and lasting effects of early trauma, implementing trauma-informed care is vital. Early identification and targeted therapeutic strategies may significantly improve emotional regulation in the long term and interrupt the entrenchment of BPD traits. Further research is needed to clarify how early maltreatment disrupts multiple aspects of child development and contributes to the chronic course of BPD. Our conceptual summary outlines thematic pathways rooted in the literature. It emphasizes areas where current research is lacking, particularly in addressing invisible trauma, considering these pathways also aid in guiding trauma-informed approaches by building skills and modeling principles that align with the social learning theory.
